# Nutritive Value Response of Native Warm-Season Forage Grasses to Harvest Intervals and Durations in Mixed Stands

**DOI:** 10.3390/plants3020266

**Published:** 2014-05-16

**Authors:** Vitalis W. Temu, Brian J. Rude, Brian S. Baldwin

**Affiliations:** 1 Agricultural Research Station, Virginia State University, Petersburg, VA 23806, USA; 2 Animal and Dairy Science Department, Mississippi State University, Mississippi State, MS 39762, USA; E-Mail: BRude@ads.msstate.edu; 3 Plant and Soil Sciences Department, Mississippi State University, Mississippi State, MS 39762, USA; E-Mail: BBaldwin@pss.msstate.edu

**Keywords:** native grass, warm-season, bluestem, digestibility, composition, crude protein, defoliation, forage quality, mixed stand, harvest interval

## Abstract

Interest in management of native warm-season grasses for multiple uses is growing in southeastern USA. Forage quality response of early-succession mixed stands of big bluestem (BB, *Andropogon gerardii*), indiangrass (IG, *Sorghastrum nutans*), and little bluestem (SG, *Schizachyrium scoparium*) to harvest intervals (30-, 40-, 60-, 90 or 120-d) and durations (one or two years) were assessed in crop-field buffers. Over three years, phased harvestings were initiated in May, on sets of randomized plots, ≥90 cm apart, in five replications (blocks) to produce one-, two-, and three-year-old stands, by the third year. Whole-plot regrowths were machine-harvested after collecting species (IG and LB) sample tillers for leafiness estimates. Species-specific leaf area (SLA) and leaf-to-stem ratio (LSR) were greater for early-season harvests and shorter intervals. In a similar pattern, whole-plot crude protein concentrations were greatest for the 30-d (74 g·kg^−1^ DM) and the least (40 g·kg^−1^ DM) for the 120-d interval. Corresponding neutral detergent fiber (NDF) values were the lowest (620 g·kg^−1^ DM) and highest (710 g·kg^−1^ DM), respectively. *In vitro* dry matter and NDF digestibility were greater for early-season harvests at shorter intervals (63 and 720 g·kg^−1^ DM). With strategic harvesting, similar stands may produce quality hay for beef cattle weight gain.

## 1. Introduction

In the southeastern USA, frequent summer forage shortages have aroused a growing interest in native warm-season grasses (NWSG) as alternative forage resources [1]. Favored NWSG species include; indiangrass (IG, *Sorghastrum nutans* (L.). Nash), big bluestem (BB, *Andropogon gerardii* Vitman), little bluestem (LB, *Schizachyrium scoparium* (Michx). Nash). switchgrass (*Panicum virgatum* L.) and eastern gamagrass (*Tripsacum dactyloides* L.) [2]. Most NWSGs are considered valuable summer forage resournces for ruminants [3,4] since they can grow well at elevated temperatures to produce digestible biomass [4]. However, their nutritive value changes with defoliation management [5] and information on NWSGs in mixed stands is scarce.

Usually, forage nutritive value of a plant can be predicted based on leafiness, which varies positively with crude protein (CP) concentration and digestibility [6]. For grasses, leafiness usually declines towards plant maturity, due to factors like shuttering of senescent dry leaves and translocation of leaf carbohydrates to the crown and roots. Forage nutritive value assessment can also include such attributes as leaf-to-stem ratio (LSR), based on weight and species-specific leaf area (SLA), which correlates negatively with leaf-fiber content and positively with digestibility [6]. Other indicators of forage nutritive value are types and concentration of: neutral detergent fiber (NDF)—containing hemicellulose, cellulose and lignin—acid detergent fiber (ADF)—with its high levels of cellulose and lignin—and acid detergent lignin (ADL)—lignin—which is an indigestible herbage component [7]. Together, they affect total forage dry matter (DM) intake and digestibility [8,9]. For a percentage increase in lignin, digestible DM decreases by three to four units [9]; this is true for both native and introduced grasses [10]. Unfortunately, most warm-season grasses have short-lived active vegetative growth [11,12] and are early maturing. As grasses mature, they grow reproductive tillers with more proportions of structural carbohydrates and phenolic compounds, mainly cellulose and lignin [3,10], while CP decreases [12]. At maturity, LSR also decreases while the proportion of senescent leaves increases [9,12].

Although most NWSGs get more lignified and less digestible in late-summer, they still make quality forages when cut at early stages [11,13]. Even at the same phenological stage, recovering defoliated plants usually have more nutritious leaves than their undefoliated counterparts [14]. This happens as the recovering plants preferentially allocate more reserve carbohydrates to leaf growth ahead of roots and reproductive structures [14,15]. In mixed stands, however, forage nutritive value of NWSGs is greatly influenced by how the dominant species respond to management. There is lack of information on how yield and forage quality of mixed stands dominated by BB, IG, and LB may respond to haying regimes. Knowing how yield and forage quality of these NWSGs, in mixed stands, may respond to harvesting for consecutive years will be useful to producers making informed management decisions. However, the objectives of this component of the study, focused on the effects of harvest interval and duration on forage nutritive value of early-succession mixed-stands dominated by BB, IG, and LB in terms of chemical composition, *in vitro* digestibility, and species morphological components.

## 2. Results and Discussion

### 2.1. Chemical Composition of Forage Dry Matter

To determine harvest interval effects on forage nutritive value, mean concentrations of CP, NDF, ADF, and ADL in whole-plot forage samples were compared by harvest interval, harvest date, and number of years in production. The comparison only involved data from June–September regrowth harvests after the first equalizing May harvest. The May harvest data were excluded from the analyses since fields are not usually harvested for forage this early.

#### 2.1.1. Crude Protein Concentration

Forage nutritive value assessment by CP concentration in DM was based on the recommended minimum of 70 g·kg^−1^ DM for beef cattle [16]. Harvest intervals, harvest year, and number of years in production did not interact to affect CP concentration, so means were pooled across harvest years and years in production for comparison (Table 1). For the second harvest after mid-May (the only one with five entries), CP concentrations were lower (42 g·kg^−1^ DM) for the 120-d harvest interval (*p* < 0.01) compared with the 30-d and 40-d (74 g·kg^−1^ DM). A similar trend was also observed for the third and fourth harvests. Except for the 60-d harvest interval, CP concentration values decreased across the season to the lowest in the fifth harvest of the 30-d (47 g·kg^−1^ DM) and fourth of the 40-d (52 g·kg^−1^ DM) harvest intervals.

**Table 1:** Effects of harvest interval and harvest date on forage nutritive value based on crude protein (CP) concentration in dry matter (DM) after the first harvest (mid-May) from mixed native grass stands ^†^ pooled across two harvest years and durations.

Frequency	Harvested regrowth				*p* > Fα ^§^
	2nd	3rd	4th	5th	
	g·kg^−1^ DM				
120 (2) ^‡^	42 E				
90 (2)	44 DE				
60 (3)	54 C	52 CD			0.54
40 (4)	69a ^#^ AB	64a B	52b CD		<0.01
30 (5)	74a A	67ab AB	63b B	47c CDE	<0.01

^†^ Stands of indiangrass (*Sorghastrum nutans*), big bluestem (*Andropogon gerardii*) and little bluestem (*Schizachyrium scoparium*). ^‡^ Days between successive harvests with number of harvests per season in brackets. ^§^ Probability of difference between means within a row. Means followed by different upper case letters differ significantly. ^#^ Means, within a row, followed by different lower case letters differ. Mean differences declared significant at α = 0.05.

The CP concentration values were between reported averages of 97 g·kg^−1^ DM (leaf) and 43 g·kg^−1^ DM (stem) for big bluestem and switchgrass at early head emergence [3]. In part, these trends were probably influenced by the fact that CP in the current study was measured on mixed whole-plot samples, and that proportions of broadleaf material in subsequent harvests decreased. The observed lower CP concentrations when days to the second harvest were longer (Table 1), portrayed a common phenomenon associated with cell wall lignification towards maturity to ensure structural support to leaves and inflorescence [15]. This would generally increase cell wall proportions while decreasing cell contents in stems and leaves. For example, in five weeks, cell contents of bermudagrass can decrease from 65% to 40% and CP concentration from 120 to 80 g·kg^−1^ DM, as that of cell walls increases from 35% to 65%, respectively [4]. Similarly, leaf CP concentration of big bluestem declines for prolonged regrowth periods [5].

**Figure 1:**
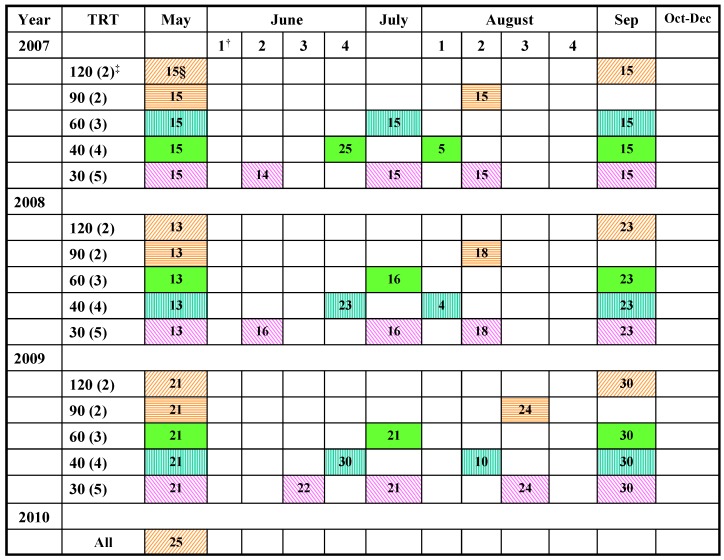
Actual harvest days by harvest interval over the experimental period.

The observed decrease in late-season CP concentrations could also be due to changes in environmental factors, mainly temperature, solar radiation, soil moisture, and nutrient availability [12]. Even at the same age, forage nutritive value usually decreases as temperature increases [17]. Lower CP concentration in late-season harvests for grasses also results from temperature- and photoperiod-induced transition into the reproductive phase, which is usually richer in stem components [18]. For the 30-d harvest interval, this transition might have coincided with the fourth harvest, August 15 (Figure 1). Comparable declines in CP concentration values from 110 g·kg^−1^ DM (May) to only 20 g·kg^−1^ DM (beyond September), have also been reported [19]. Additionally, most harvesting events in the current study were followed by heavy rains, which usually leach soil nitrogen [20] and thus reduce CP concentration in the biomass.

#### 2.1.2. Across-Season Crude Protein Fluctuations

To identify possible combinations of harvest intervals and dates for good quality hay, CP concentrations of independent harvests, pooled across harvest-years and number of years in production were compared (Table 1). The CP values for the first (74 g·kg^−1^ DM) and second (67 g·kg^−1^ DM) harvests of the 30-d and the first (69 g·kg^−1^ DM) of the 40-d interval were similar and ranked higher than the third of 30-d (63 g·kg^−1^ DM) and second of 40-d (64 g·kg^−1^ DM). However, only values in the higher rank met or closely approached the minimum of 70 g·kg^−1^ DM for quality hay [16]. Other values (<55 g·kg^−1^ DM) were far below this minimum. Similarly, the second harvest of the 40-d interval with 67 g·kg^−1^ DM CP (nearly 70) could also make good-quality hay. This ranking showed that both timing and days between successive harvests have implications on hay quality, which is compromised by a >10-d delay, for the 30-d intervals.

#### 2.1.3. Forage Fiber Concentration

Nutritive value assessment also involved comparing the proportions of NDF, ADF, and ADL in the DM samples (Table 2) because they negatively affect intake and digestibility [21]. Results on fiber concentration in the whole-plot forage samples are discussed with reference to their respective desirable levels to support weight gains of beef cattle.

##### 2.1.3.1. Neutral Detergent Fiber (NDF) Concentration

**Figure 2:**
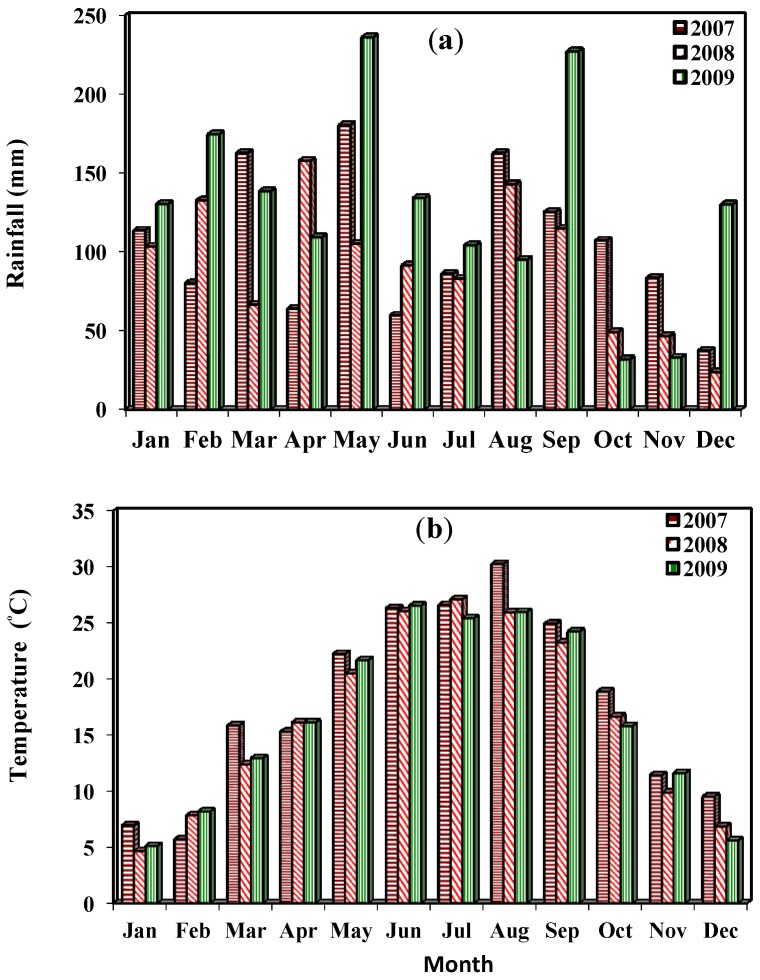
(**a**) Temporal trends in monthly rainfall totals (mm); (**b**) Trends in monthly mean temperature (°C) during the study period, 2007 to 2009, Aberdeen, MS.

Though NDF is partially digestible by ruminants, the extent varies between plant species and maturity stage [22]. Usually, grass hay with 550 g·kg^−1^ DM to 600 g·kg^−1^ DM NDF concentration is of good quality, while those with 700 to 800 g·kg^−1^ DM are considered poor in quality [23]. This is so because NDF concentration is inversely related to voluntary DM intake [20]. The ADF fraction is of less digestible fiber and is negatively correlated with intake [24] which should not exceed 300 to 350 g·kg^−1^ DM for good-quality grasses [23]. Within the 30-d harvest interval, there were year differences in relation to NDF concentrations which could be attributed to changes in weather conditions (Figure 2) and a 5-d delay of the second harvest (22 June) in 2009 (Figure 1). In 2008, NDF values only differed between the 30-d and 90-d harvest intervals and between the 60-d harvests (Table 2). In 2009, however, NDF concentrations had lesser values for the second, compared to later harvests in the season (*p* < 0.03). For the second harvest of the 30-d, 40-d, and 60-d intervals, NDF values were similar and slightly above the upper limit of 600 g·kg^−1^ DM for high-quality forage, but far below 700 g·kg^−1^ DM to be considered poor-quality ones [23]. Even as the season advanced, NDF values remained about 670 g·kg^−1^ DM, which tends to be of poor-quality hay.

**Table 2:** Effects of harvest interval on forage quality, based on neutral detergent fiber (NDF), acid detergent fiber (ADF), and acid detergent lignin (ADL) concentrations in the dry matter for each harvest, after the first (mid-May) from mixed native grass stands ^†^ in 2008 and 2009 harvest-years pooled over two harvest durations ^‡^.

Interval (days)	June-September 2008					June-September 2009				
	2nd	3rd	4th	5th	*p* > Fα ^§^	2nd	3rd	4th	5th	*p* > Fα
	g·kg^−1^ DM					g·kg^−1^ DM				
NDF										
120 (2)	670 ABC ^#^	-	-	-	-	710 A	-	-	-	-
90 (2)	650 C	-	-	-	-	660 B	-	-	-	-
60 (3)	680a AB	650b	-	-	<0.02	640b BC	680a	-	-	<0.01
40 (4)	660 BC	650	630	-	0.33	630b BC	660ab	670a	-	<0.02
30 (5)	690 A	670	670	670	0.08	620b C	670a	670a	660a	<0.03
ADF										
120 (2)	360	-	-	-	-	400 A	-	-	-	-
90 (2)	360	-	-	-	-	360 B	-	-	-	-
60 (3)	370	360	-	-	0.41	340b BC	370a	-	-	<0.01
40 (4)	350	350	350	-	0.90	330c C	350b	370a	-	<0.01
30 (5)	380	360	380	370	0.13	340b BC	370a	330b	380a	<0.01
ADL										
120 (2)	54 A	-	-	-	-	52 A	-	-	-	-
90 (2)	53 A	-	-	-	-	47 AB	-	-	-	-
60 (3)	51 AB	58	-	-	0.19	37 C	43	-	-	0.14
40 (4)	37b C	41b	55a	-	<0.011	43a BC	31b	46a	-	<0.01
30 (5)	42c BC	48bc	53b	67a	<0.01	46 AB	44	44	45	0.9

^†^ Stands of indiangrass (*Sorghastrum nutans*), big bluestem (*Andropogon gerardii*) and little bluestem (*Schizachyrium scoparium*). ^‡^ Combined data from plots harvested for the first or second time in the year. ^§^ Probability of difference between means within a row, in the same year. Days between successive harvests with number of harvests per season in brackets. ^#^ Means followed by different letters; uppercase within column or lowercase within row differ significantly at α = 0.05.

Greater NDF concentrations for longer harvest intervals are usually associated with growth of reproductive tillers and increased proportions of structural carbohydrates for physical support [8,10,12]. Greater NDF concentration in late-season harvests is usually a phenological response to hot temperatures and short day lengths, which induces faster development with reduced LSR [25]. Similar findings have been reported for switchgrass with NDF concentration at maturity, averaging 640 g·kg^−1^ DM in May, but up to 790 g·kg^−1^ DM in September and beyond [19]. Measured NDF concentrations could also be influenced by changes in types and proportions of annual forbs in the stands across the season due to defoliation and weather conditions. In fact, a notable increase in short-growing annual grasses and forbs was associated with the 30-d harvest interval (data not included) although the proportions of BB, IG, and LB in the stands were not altered.

##### 2.1.3.2. Acid Detergent Fiber (ADF) Concentration

Differences in ADF concentration between harvest intervals or harvest dates within a season were not observed in 2008 (Table 2), but were in 2009. As with NDF, ADF concentrations in the second harvest after mid-May of 2009 were greater for longer intervals, which suggests increased lignification of the cell walls at maturity. These ADL values were consistent with their corresponding decrease in CP concentrations. However, within harvest interval, and across the season, ADF concentrations for the 30-d were lesser at the second and fourth harvests, averaging 335 g·kg^−1^ DM, but greater at the third and fifth (375 g·kg^−1^ DM). At the second and fourth harvests of the 30-d interval, ADF values were actually below the upper limit of 350 g·kg^−1^ DM for good-quality hay [23]. This pattern of changes in ADF concentrations also matched the unusual weather conditions, in 2009 (Figure 2a,b), with heavy rainfall in May and September and a prolonged hot June–August dry spell. It seems that elevated temperatures, in 2009, created drought conditions towards the third and fifth harvests, which usually result with reduced plant size and less lignified tissues [25].

##### 2.1.3.3. Acid Detergent Lignin (ADL) Concentration

Lignin, an anti-quality component deposited in plant cell walls at maturity [17], is thought to interfere with fiber digestion. Such interference may involve obstructing microbial enzymes [26], or forming chemical cross-linkages with fiber polysaccharides [12]. Therefore, forages with lower ADL concentrations are more desirable. In this study, ADL data showed effects due to harvest year, harvest intervals and dates across the season (Table 2). During both the 2008 and 2009 seasons, ADL concentrations for the second harvest, after mid-May, were about 40 g·kg^−1^ DM at shorter harvest intervals and increased to slightly above 50 g·kg^−1^ DM for the 120-d. However, year differences were also observed in ADL concentrations, across the season, with the greatest value (67 g·kg^−1^ DM) being for the fifth harvest in 2008, while in 2009 differences were only observed between the 40-d harvests.

The observed greater ADL concentrations at longer harvest intervals were characteristic of mature forage plants [17]. However, there were some discrepancies between values from this study and those in the literature. For example, while some values were above a reported pooled average of 47 g·kg^−1^ DM for big bluestem and switchgrass leaf tissues [3], most values were below this average. Still all values came below a reported 56 g·kg^−1^ DM for big bluestem [4]. Yet, all were mostly above the range of 34 to 43 g·kg^−1^ DM for mixed warm-season grasses [27]. The noted discrepancies could be explained by the fact that ADL values in the current study were of mixed stands, whose biomass was not sorted by species, or leaf and stem components. As in the case of NDF above, increased proportion of annuals in the biomass at the 30-d harvest interval, likely influenced changes in ADL concentrations in the succeeding harvest samples.

### 2.2. Forage in Vitro Digestibility

As another indicator of forage quality to ruminants, *in vitro* digestibility values of the DM (IVDMD) and NDF (NDFD in the harvested material were compared for effects of harvest interval and harvest dates across the season, in two consecutive years. The results (Table 3) are discussed in two subsections.

#### 2.2.1. Harvest Interval and DM Digestibility

Usually, grasses with a minimum of 550 g·kg^−1^ IVDMD are considered good quality forages [19]. This is necessary information when estimating potential DMI by animals, which is usually influenced by how easily digestible the forage is. Significant year-effects on measured IVDMD were observed, so mean comparison results are presented by year (Table 3). Effects of harvest interval or harvest date on IVDMD were not observed in 2008 and values averaged 460 g·kg^−1^ DM. In 2009, IVDMD values for the second harvest were above 600 g·kg^−1^ DM for the 30-d and 40-d intervals and decreased for the longer harvest-intervals to 450 g·kg^−1^ DM in the 120-d.

The observed lower digestibility values for longer harvest intervals were likely a result of: (1) Most tillers being harvested at their transition into reproductive phase, usually characterized by jointed stems and lignified cell walls [25]; (2) Translocation of carbohydrates to the crown and roots, that left standing biomass richer in cell walls than cell contents; and (3) Shuttering of senescent leaves at harvest, which usually increases the proportion of stems in the biomass [8]. These results are similar to reported declines in IVDMD from 520 and 450 g·kg^−1^ DM, for big bluestem shoots, clipped at 30, 41, and 51 cm stages [5]. Similar declines in DM digestibility from 708 to 566 g·kg^−1^ DM between late vegetative and early heading stages have also been reported on mixed switchgrass and big bluestem hay [11]. On the other hand, greater digestibility values for shorter harvest intervals were also consistent with the observed decrease in fiber concentrations (NDF, ADF and ADL) discussed above. Plant biomass with less fiber is usually less lignified and therefore easily digested [13,14]. In the current study, differences in DM digestibility indicate greater availability of nutrients in the native grasses harvested at early rather than late-growth stages.

**Table 3:** Effect of harvest interval on *in vitro* dry matter (DM) and neutral detergent fiber (NDF) digestibility for each harvest after the first (mid-May) from mixed native grass stands ^†^ in 2008 and 2009 harvest-years pooled over two harvest durations ^‡^.

Interval (days)	June–September 2008					June–September 2009				
	Harvest Timing				*p* > Fα ^§^	Harvest Timing				*p* > Fα
	2nd	3rd	4th	5th		2nd	3rd	4th	5th	
	g·kg^−1^ DM					g·kg^−1^ DM				
DM										
120 (2)	420	-	-	-	-	450 D ^#^	-	-	-	-
90 (2)	470	-	-	-	-	530 C	-	-	-	-
60 (3)	460	480	-	-	0.48	580a ^††^ BC	490b	-	-	<0.01
40 (4)	490	510	470	-	0.26	650a A	570b	480c	-	<0.01
30 (5)	440	440	430	430	0.87	630a AB	540b	640a	500b	<0.01
NDF										
120 (2)	560 C	-	-	-	-	550 D	-	-	-	-
90 (2)	600 BC	-	-	-	-	650 C	-	-	-	-
60 (3)	620 AB	620	-	-	0.93	680a BC	600b	-	-	<0.01
40 (4)	670a A	650ab	610c	-	0.02	740a A	670b	590c	-	<0.01
30 (5)	620 AB	620	590	570	0.11	720a AB	650b	740a	600b	<0.01

^†^ Stands of indiangrass (*Sorghastrum nutans*), big bluestem (*Andropogon gerardii*) and little bluestem (*Schizachyrium scoparium*). ^‡^ Combined data from plots harvested for the first or second time in the year. ^§^ Probability that, in the same year, means within a row differed significantly. Days between successive harvests, with number of harvests per season in brackets. ^#^ Means followed by different letters; uppercase within column or lowercase within row, are different. Mean differences declared significant at α = 0.05.

#### 2.2.2. Harvest Timing and DM Digestibility

To assess the influence of weather-induced phenological changes on forage quality indicators, IVDMD values were compared between harvest dates, within harvest intervals (Table 3). For the 30-d harvest interval, IVDMD values of the second and fourth harvests were similar (>600 g·kg^−1^ DM) and greater (*p* < 0.01) than those of the third and fifth harvests by about 100 units (Table 4). Within the 40-d harvest interval, IVDMD was greater for the second ones (650 g·kg^−1^ DM) than the third (570 g·kg^−1^ DM) and fourth (480 g·kg^−1^ DM) harvests (*p* < 0.01). These IVDMD values are similar to others in literature for leaves (604 g·kg^−1^) and stems (500 g·kg^−1^) of mixed big bluestem and switchgrass at early head emergence [3,28]. Dry matter digestibility of big bluestem hay has also been found to decline from 678 to 546 g·kg^−1^ between late July and early August [11]. Although all IVDMD values for the 30-, 40-, and 60-d harvest intervals were well above the minimum of 550 g·kg^−1^ for quality forage [16], issues of acceptability associated with NDF concentration would make the 60-d harvests undesirable.

**Table 4:** Effects of harvest interval and harvest duration on mean June–September leaf:stem ratio (LSR) and specific leaf area (SLA) of indiangrass (*Sorghastrum nutans*) and little bluestem (*Schizachyrium scoparium*) tillers from mixed native grass stands ^†^ at their first and second harvest year recorded at each harvest after the first (mid-May) in 2008 and 2009.

Interval (days)	LSR				SLA			
	2008		2009		2008		2009	
	Y108 ^‡^	Y207	Y109	Y208	Y108	Y207	Y109	Y208
					cm^−2^·g^−1^			
Indiangrass								
Control	-	-	0.4b ^§^	0.4c	-	-	98d	98d
120 (2)	1.6	1.9	0.7b	0.8bc	109c	102b	101d	110d
90 (2)	1.8	1.3	0.8b B	1.4ab A	107c	100b	108d	110d
60 (3)	1.7	1.7	1.5a	1.4ab	112c	117b	227c	243c
40 (4)	2.1	2.1	1.4a B	1.8a A	137b	150a	403b	368b
30 (5)	1.8	2.0	1.3a B	1.9a A	152a	154a	567a A	529a B
*p* > F_α_^#^	0.57	0.13	<0.01	<0.01	<0.01	<0.01	<0.01	<0.01
Little bluestem								
Control	-	-	0.4b	0.4c	-	-	118d	118d
120 (2)	1.2	1.0	0.6b	0.7bc	132b	134c	119d	128d
90 (2)	1.3	2.1	0.8b B	1.2ab A	133b	136c	131d	156d
60 (3)	1.5	1.6	1.2a B	1.5a A	162a	156bc	284c	306c
40 (4)	1.7B	1.9A	1.3a	1.2ab	168a	166ab	475ab	470b
30 (5)	1.9	2.3	1.4a	1.5a	162a	194a	732a A	674a B
*p* > F_α_	0.14	0.21	<0.01	<0.01	<0.01	<0.01	<0.01	<0.01

^†^ Stands of indiangrass (*Sorghastrum nutans*), big bluestem (*Andropogon gerardii*) and little bluestem (*Schizachyrium scoparium*). ^‡^ Y108, Y109, Y207 and Y208 are plots in their first and second harvesting year, established in 2008, 2009 and 2007, respectively. ^§^ Means of the same attribute followed by different letters; lowercase within column or uppercase within row differ. Days between successive harvests with number of harvests per season in brackets. ^#^ Probability that, means in respective columns differ significantly at α = 0.05.

It is also likely that increased rainfall and warm temperatures in June of 2009 induced faster plant development, similar to earlier findings [25]. Such growing conditions often result with reduced LSR and increased forage fiber, characteristic of less digestible biomass. The fact that weather conditions in June were generally reproduced in August may also explain the observed patterns of forage IVDMD values. It appears that forage nutritive value in 2009, for the third harvest at the 30-d interval, was more influenced by the prolonged June–August dry spell (Figure 1). Usually, drought stresses, when not severe enough to kill plants, tend to increase their forage digestibility [20]. This is so because affected plants grow less vigorously with less lignified cell walls due to reduced demand for mechanical support [25]. However, most IVDMD values at the 30- and 40-d harvest intervals (Table 4) were above or very close to 550 g·kg^−1^ that is recommended for good quality forages [16,20]. Therefore, management interventions intended to boost biomass production under similar conditions should target the early-season harvests at 30- to 40-d intervals.

#### 2.2.3. Harvest Interval on NDF Digestibility

Neutral detergent fiber digestibility values were in patterns similar to those of IVDMD (Table 3) indicating that forage quality was better for the early-season biomass at shorter harvest intervals. In 2008, the second harvest at the 40-d interval had greater NDFD (670 g·kg^−1^ DM) than the 90-d (600 g·kg^−1^ DM) and 120-d (560 g·kg^−1^ DM) harvests, but not different from the 30-d and 60-d (620 g·kg^−1^ DM). In 2009, NDFD for the first regrowth of the 40-d harvest interval (740 g·kg^−1^ DM) was greater than for the 60-d (680 g·kg^−1^ DM), 90-d (650 g·kg^−1^ DM) and 120-d (550 g·kg^−1^ DM), but not different from the 30-d harvests (720 g·kg^−1^ DM). The observed lower NDFD at longer harvest intervals was similar to reported decline in grass NDFD of up to 400 g·kg^−1^ DM due to maturity alone [29]. This suggests that fiber lignin concentrations increased as more plants in the mixed stands matured. Such declines in forage nutritive value are usually associated with developmental changes. These changes usually involve development of xylem vessels for water transport, accumulation of cellulose and complex carbohydrates, all bound together by lignin deposition [26]. Such changes would make plant cell walls less digestible.

#### 2.2.4. Harvest Timing on NDF Digestibility

Means, for both 2008 and 2009 data, were also compared between harvest dates, within harvest intervals (Table 3). In 2008, effects of harvest date on NDFD were only significant for the 40-d harvest interval, being greater (*p* < 0.03) at the second harvest (670 g·kg^−1^ DM) than the fourth (610 g·kg^−1^ DM), but not the third (650 g·kg^−1^ DM). As was for IVDMD in 2009, NDFD within the 30-d harvest interval was not different between the second and fourth harvests which averaged above 700 g·kg^−1^ DM. These were also greater (*p* < 0.01) than 625 g·kg^−1^ DM for the third and fifth harvests. Within the 40-d harvest interval, NDFD was 740 g·kg^−1^ DM for the second harvest and greater than the third (670 g·kg^−1^ DM) and fourth (590 g·kg^−1^ DM). With mixed grasses, NDFD values have been found to decline from 800 g·kg^−1^ DM for early-May harvest to 440 g·kg^−1^ DM by late-June while lignin concentrations increased from 17 to 53 g·kg^−1^ DM, respectively [7]. However, most NDFD values for the 30- and 40-d harvest intervals were between 650 and 540 g·kg^−1^ DM, recommended for good- to medium-quality grass hay, respectively [30]. This implies that increased NDF concentration in the late-season harvests might not severely limit DMI, owing to their greater digestibility values. Decline in NDFD is usually a result of amounts and types of lignin deposited, which differ between species [26]. Additionally, forbs in the current study appeared to account for a substantial proportion of the regrowth biomass, which possibly improved the observed average digestibility.

### 2.3. Species Tiller Leafiness

How harvest intervals or number of consecutive harvest years might affect species forage quality was also assessed on two morphological components associated with leafiness. This was based on the fact that leafiness of plant material, expressed as LSR or SLA, is a common indicator of forage nutritive value [6]. For both IG and LB, in 2008 and 2009 harvest years, changes in LSR and SLA due to harvest dates, within harvest interval, were rare and inconsistent (Table 4). Effects of harvest interval and harvest duration were, therefore, assessed on their respective June–September averages.

#### 2.3.1. Tiller Leaf-to-Stem Ratio

In 2008, IG showed no effect of harvest interval or harvest duration (number of years in production) on LSR, which averaged 1.8 (Table 4). Similarly, LSR for LB was not affected by harvest interval and values ranged from 1.5 to 1.8 for plants in their first and second year of production, respectively. Only the 40-d harvest interval had greater LSR (1.9) for the second-year plants. In 2009, means of LSR, within harvest interval, differed (*p* < 0.01) between plants in first (Y109) and second (Y208) year of production. For Y109 plots, harvesting IG at <90-d intervals resulted with greater LSR (1.5) for the 60-d harvest interval, almost 4-fold the 0.4 at first harvest (control). Still, IG harvested at <120-d intervals had greater LSR in the second- (Y208) rather than in first-year (Y109) plots. A similar trend was observed for LB whose LSR values for the control plants (0.4) were lower (*p* < 0.01) by more than 50% compared to plants harvested at intervals ≤90-d, which ranged from 0.8 to 1.5 (Table 4).

The absence of harvest interval effect on tiller LSR in 2008 was likely due to adequate rainfall distribution (Figure 1a) that possibly allowed compensatory growth to override the negative effects of defoliation. Having greater LSR (*p* < 0.01) for shorter harvest intervals in 2009 suggests that respective harvest events mostly coincided with vegetative stage of the regrowth, usually characterized by faster growth of leaf blades than stems and leaf sheaths [15,21]. The decreased LSR values observed for the 90-d and 120-d harvest intervals were also characteristic of assimilate translocation to the crown and shuttering of senescent dry leaves [21]. These LSR values also reflected late-season changes in temperature and photoperiod, which usually prompt grasses to transition into reproductive phase with reduced LSR [18]. Hence grasses at the reproductive phase would make poor hay even at short harvest intervals. Greater LSR values for the second- rather than first-year plants were expected because defoliation often results with thinner tillers, mostly vegetative, and with more leaves than stems [31].

#### 2.3.2. Tiller Specific Leaf Area

The SLA of IG in the first and second year of production was affected (*p* < 0.01) by harvest interval during both 2008 and 2009 (Table 4). In 2008, SLA ranged from <110 cm^−2^·g^−1^ for plants harvested at intervals ≥90-d to >135 cm^−2^·g^−1^ for harvesting at <60-d intervals. While the control plants and those harvested at a >60-d interval in 2009 had SLA (104 cm^−2^·g^−1^) comparable to that of 2008; SLA for plants harvested at a 30-d interval reached over 500 cm^−2^·g^−1^ (Table 1). These SLA values were greater for plants in their first rather than second year of production by over 35 units. In similar trends, SLA of LB in Y108 and Y207 plots was greater (*p* < 0.01) for plants harvested at shorter intervals (Table 1). Within harvest intervals, SLA across years of production, in 2008, ranged from 133 cm^−2^·g^−^^1^ (120-d) to 176 cm^−2^·g^−1^ (30-d) with no difference between Y108 and Y207 plots (Table 4).

In 2009, SLA of LB across years of production for the control plants and those harvested at a >60-d interval averaged 128 cm^−2^·g^−1^. However, values for plants harvested at shorter intervals were greater than 250 cm^−2^·g^−1^ and reached well over 700 cm^−2^·g^−1^ (Y109) and 650 cm^−2^·g^−1^ (Y208) for the 30-d interval (Table 4). The observed greater SLA values for shorter harvest intervals are characteristic of vegetative growth phase of grasses, at which leaf blade elongation and expansion rates exceed that of stems [21]. At longer harvest intervals, plants are more likely to transition into the reproductive phase during which growth is more of stem elongation and cell wall lignification [15,21] consistent with observed increase in fiber content. These observed changes in tiller LSR and SLA values indicated that the native grasses produced better quality forage when harvested at shorter rather than longer intervals. These results on species morphological components, therefore, further stress the importance of timely harvesting for similar mixed stands dominated by IG and LB to produce quality hay.

## 3. Experimental Section

### 3.1. Study Location and Field Layout

**Figure 3:**
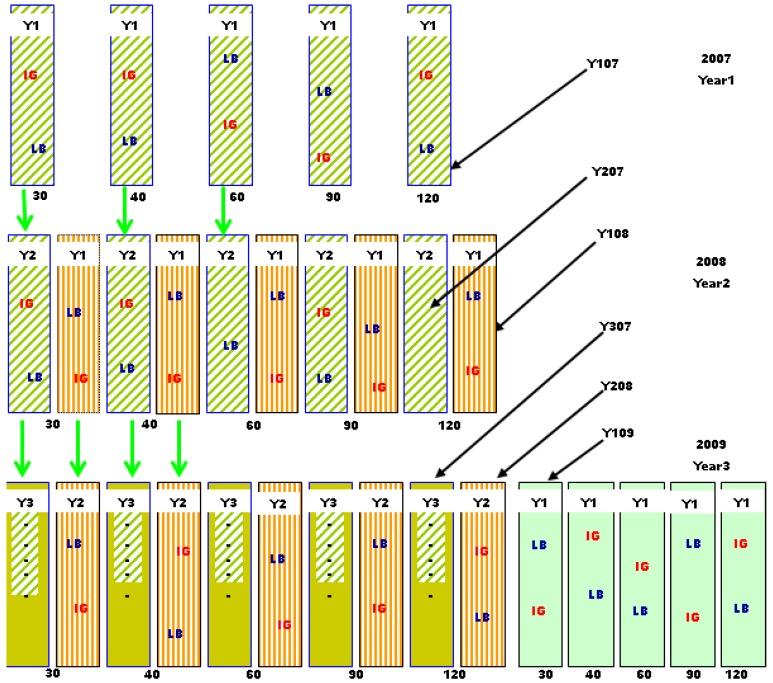
Plot arrangement, in one replication, showing establishment sequence. Five first-year plots (Y1) established in mid-May from 2007 to 2009, each 7.5 m long and 1 m wide, with marked and monitored indiangrass and little bluestem plants assigned to 30-, 40-, 60-, 90-, and 120-d harvest intervals. In each year, plots are labeled Y1, Y2, or Y3 indicating plots beginning their first, second and third harvest year, respectively.

This study was conducted at Bryan Farms, Clay County, (33°39'N; 88°34'W) MI, USA, in unfertilized conservation field buffers planted with mixed NWSGs, at their early-succession stages. Dominant soils in the study area are Griffith silty clay, classified as Fine, smectitic, thermic Aquic Hapludert with pH ranging from 5.0 to 5.6 and Okolona silty clay, classified as Fine, smectitic, thermic Oxyaquic Hapludert with pH range of 6.0 to 7.8. A seed mixture of 1.12 kg BB, 2.24 kg LB, and 1.12 kg IG per hectare of prepared seedbed was sown in 2005, and allowed to grow undisturbed for two years. Extended post-emergence herbicide (imazapic at 0.28 kg a.i ha^−1^) {(±)-2-[4,5-dihydro-4-methyl-4-(1-methylethyl)-5-oxo-1H-imidazol-2-yl]-5-methyl-3-pyridinecarboxylic acid} was applied to control competitive weeds. In late spring of 2007, five 7.5 × 1-m parallel strips, at least 3 m apart were randomly assigned to five, four, and three harvests at 30-, 40-, and 60-d intervals, respectively, or only two harvests at 90- or 120-d interval (Figure 3), giving five harvest intervals per block. The 90-d interval mimicked a standard practice of harvesting a hay crop early in the growing season, and then stockpiling the regrowth for late-season grazing or conservation uses. In a randomized complete block design, these five harvest intervals were replicated in five blocks, three in two buffers of one crop field and two in another field, about 5 km away, on similar soils.

During the spring of 2008, other 7.5 × 1-m plots were marked next to each previous-year plot with 90-cm alleys between the first- and second-year plots for each harvest interval. Plots harvested first in 2007 were designated Y207, indicating they were in their second harvest year (Y2), but started in 2007 (07). Plots harvested first in 2008 adjacent to Y207 plots were designated Y108, indicating they were in their first harvest year (Y1), but started in 2008 (08). In 2009, a third set of five 7.5 × 1-m, plots separated by 90-cm alleys were marked on one end of each block; a total of three plots per harvest interval per block. Adding the third set of plots on the respective block ends was necessary to avoid possible negative effects of the two-year feet and machine traffic on plant growth. For each block, however, an area with relatively uniform species composition, terrain, and plant vigor, large enough to accommodate all three sets of plots, was clearly defined in the first harvest-year. With this arrangement, there were no notable differences in plant performance between third year plots and the rest, within a harvest interval. Plots started in 2009, were designated Y109 while the Y108 plots re-designated Y208 and the Y207 became Y307 (Figure 3). In spring of 2009, the Y307 plots were harvested only once, in May, to assess post-season recovery and then removed from the harvest regime. To avoid shedding, plants in the separating alleys next to harvested plots were also trimmed to the same height, using a hand-held weed eater on each harvested day.

### 3.2. Harvesting and Forage Sampling

In mid-May of each year, all study plots received a common/equalizing harvest, after which regrowth was harvested on assigned dates throughout the summer (Figure 2). Occasionally, harvesting was hastened by one to two, or delayed for up to six days (Figure 2) to avoid major rainfall events, thus allowing optimum machine operation. Whole-plot forage was harvested by a 1.0 m wide Carter Flail Forage Harvester (Carter Manufacturing Company, Inc., Brookston, IN, USA). At harvest, fresh whole-plot forage sample was collected from each plot and later dried in a forced-air oven at 65 °C to constant weight and processed for analyses, described below.

### 3.3. Species Morphological Assessment

Assessment of species leafiness during the growing season was based on measurements taken on tiller leaf and stem components. A day before each plot-harvest event, three tillers of IG and LB were clipped at ground level and later separated into leaves and stems by cutting through the collar, leaving leaf sheaths as stem components. Each block had a reference plot, not-harvested, from which sample tillers were collected and used as control in assessing species morphological response to the harvest intervals. In the interest of time, BB was excluded in the species assessment. Leaf blades of each tiller were run through a portable area meter (LI-COR, Model No LI-3000 LI-COR Biosciences, Lincoln, NE, USA) to determine total leaf area (LA, cm^−2^). The stem and leaf sections were then oven-dried separately at 60 °C to constant weights, cooled in desiccators and weighed on a microbalance AG 104 (Mettler-Toledo Inc., Columbus, OH, USA), to determine dry weights of tiller leaf (LW) and stem (SW) components. Tiller leaf-to-stem ratio (LSR) was calculated as LW/SW and specific leaf area (SLA, cm^2^·g^−1^) as LA/LW. For each year, LSR values, after the equalizing (mid-May) harvest, were averaged within harvest interval across harvest dates.

### 3.4. Forage Nutritive Value Assessment

Dried whole-plot samples were ground to pass a 1-mm sieve (Willey mill, Standard model 3, Arthur H. Thomas Co., Philadelphia, PA, USA) and stored in plastic sample bags until analyzed for their chemical composition and digestibility. Samples were analyzed for crude protein (CP) by block digestion method [32]. Concentrations of neutral detergent fiber (NDF), acid detergent fiber (ADF) and acid detergent lignin (ADL) in the DM as well as DM and NDF digestibility were determined according to ANKOM Technology method 3 [33]. Ash content was determined by combustion in a muffle furnace at 550 °C for four hours [33]. Percent organic matter content (DM basis) was calculated by subtracting ash content (DM basis) from 100. During each year, values for each harvesting event were averaged within harvest interval and recorded as mean sample DM, CP, NDF, *etc.*, concentration. Data for grab samples from each May harvest were handled separately.

### 3.5. Data Analyses

Data were organized and analyzed for effects of harvest interval, harvest year, and number of years in production, on forage quality attributes. The latter compared yields of first- and second-year plots assigned to a harvest interval, within a harvest year. Data were subjected to analysis of variance (ANOVA) in a randomized complete block design with harvest intervals, species, year, and harvest duration as fixed effects in five replications, using the general linear model of SAS Institute [34]. Means separation was by Fisher’s protected least significant difference (LSD) and were declared different at α = 0.05.

## 4. Conclusions

In this study, IG and LB had greater SLA and LSR for early-season tillers in the mixed stands harvested 30 and 40 days after the equalizing mid-May harvest. This showed that early-season regrowths of IG and LB under comparable conditions will take more than 30 days to begin transitioning into the reproductive phase and that after 40 days biomass increase may be more of stem than leaf elongation. Of the two, LB seemed to take longer to transition into the reproductive phase, a desirable forage quality feature. Crude protein concentrations of whole-plot forage for early-season (the second- and third) harvests at the 30-d and 40-d intervals were about the acceptable minimum of 70 g·kg^−1^ DM for good quality forage. Crude protein concentrations in whole-plot forage samples were not affected by harvest year, or number of years in production, thereby implying that forage quality of the studied native grasses, in mixed stands, may not be compromised by previous intensity of defoliation. The fact that nutritive values for the same harvest intervals change significantly after the July–August dry spell shows that drought stresses can override the forage-quality significance of harvest intervals.

There were year differences in the measured forage fiber attributes making effects of harvest intervals and date practically undetectable in the year with favorable rainfall distribution. Although measured fiber concentrations were generally indicative of good to medium quality forage—NDF, for example, ranged 600–700 g·kg^−1^ DM—their crude protein and digestibility values were limiting. This suggests that actual chemical composition of the fiber components were more responsible for observed disagreements between the measured parameters. *In vitro* DM and NDF digestibility was greater for shorter harvest intervals and fluctuated across the harvest season with greater values for the second harvest, early-season, and the regrowth coinciding with the dry spell.

Overall, data shows that mixed stands of BB, IG, and LB can produce quality hay when harvested early in the season at 30-d intervals before the summer dry spell and that a 10-d harvest delay may not compromise forage quality. Data also suggests that forage quality of NWSGs in mixed stands may be manipulated by in-season management since it was not affected by defoliation history. More studies on the effects of harvest interval and duration on individual species’ forage nutritive value aspects are needed. Similarly, feeding studies with ruminant animals to assess effects of harvest interval and duration on forage acceptability are also needed. The effects of other management practices, including spring-burning and fertilizer application, also merit further studies.
